# Mechanistic insights into the effect of imidazolium ionic liquid on lipid production by *Geotrichum fermentans*

**DOI:** 10.1186/s13068-016-0682-z

**Published:** 2016-12-16

**Authors:** Li-Ping Liu, Min-Hua Zong, Robert J. Linhardt, Wen-Yong Lou, Ning Li, Chao Huang, Hong Wu

**Affiliations:** 1 School of Food Science and Engineering, South China University of Technology, Room 409, Building 13, 381 Wushan Rd., Tianhe District, Guangzhou, 510640 China; 2 School of Bioscience and Bioengineering, South China University of Technology, Guangzhou Higher Education Mega Centre, 382 East Waihuan Rd., Panyu District, Guangzhou, 510006 China; 3 Department of Chemical and Biological Engineering, Rensselaer Polytechnic Institute, 110 Eighth Street, Troy, New York, 12180 USA; 4 Key Laboratory of Renewable Energy, Chinese Academy of Sciences, 2 Nengyuan Rd., Tianhe District, Guangzhou, 510640 China; 5 Guangdong Province Key Laboratory for Green Processing of Natural Products and Product Safety, 381 Wushan Rd., Tianhe District, Guangzhou, 510640 China

**Keywords:** Imidazolium ionic liquid, *Geotrichum fermentans*, Lignocellulosic hydrolysate, Lipid production, Inhibition

## Abstract

**Background:**

Ionic liquid (IL) pretreatment has emerged as a promising technique that enables complete utilization of lignocellulosic biomass for biofuel production. However, imidazolium IL has recently been shown to exhibit inhibitory effect on cell growth and product formation of industrial microbes, such as oleaginous microorganisms. To date, the mechanism of this inhibition remains largely unknown.

**Results:**

In this study, the feasibility of [Bmim][OAc]-pretreated rice straw hydrolysate as a substrate for microbial lipid production by *Geotrichum fermentans*, also known as *Trichosporon fermentans*, was evaluated. The residual [Bmim][OAc] present in the hydrolysate caused a reduction in biomass and lipid content (43.6 and 28.1%, respectively) of *G. fermentans*, compared with those of the control (7.8 g/L and 52.6%, respectively). Seven imidazolium ILs, [Emim][DEP], [Emim]Cl, [Amim]Cl, [Bmim]Cl, [Bzmim]Cl, [Emim][OAc], and [Bmim][OAc], capable of efficient pretreatment of lignocellulosic biomass were tested for their effects on the cell growth and lipid accumulation of *G. fermentans* to better understand the impact of imidazolium IL on the lipid production. All the ILs tested inhibited the cell growth and lipid accumulation. In addition, both the cation and the anion of IL contributed to IL toxicity. The side chain of IL cations showed a clear impact on toxicity. On examining IL anions, [OAc]^−^ was found to be more toxic than those of [DEP]^−^ and Cl^−^. IL exhibited its toxicity by inhibiting sugar consumption and key enzyme (malic enzyme and ATP-citrate lyase) activities of *G. fermentans*. Cell membrane permeability was also altered to different extents in the presence of various ILs. Scanning electron microscopy revealed that IL induces fibrous structure on the surface of *G. fermentans* cell, which might represent an adaptive mechanism of the yeast to IL.

**Conclusions:**

This work gives some mechanistic insights into the impact of imidazolium IL on the cell growth and lipid accumulation of oleaginous yeast, which is important for IL integration in lignocellulosic biofuel production, especially for microbial lipid production.

**Electronic supplementary material:**

The online version of this article (doi:10.1186/s13068-016-0682-z) contains supplementary material, which is available to authorized users.

## Background

Microbial oils, produced by oleaginous microorganisms, have garnered much interest in recent years as promising raw materials for biodiesel production due to their similarity to vegetable oils in fatty acid composition [1]. In addition, the production of microbial oils has many advantages such as short fermentation time, easy scale-up, and no required competition for agricultural land. Despite the favorable impact microbial oils might exert, the high production cost of microbial oils makes them less economically competitive. Several types of low-cost substrates, including molasses [2], wastewaters [3], and industrial glycerol [4], have been utilized to reduce the fermentation cost to make microbial oil production more competitive. Although these substrates can substantially lower production costs, their low availability make them unable to meet the increasing demand for microbial oils. Lignocellulosic biomass, the most abundant and renewable organic polymer in nature, may represent a superior substrate for the large-scale production of microbial oils.

The bioconversion of lignocellulosic biomass into microbial oils typically consists of three steps: pretreatment, saccharification, and fermentation. The pretreatment step is required due to the recalcitrance of lignocellulosic biomass to enzymatic hydrolysis. An ideal pretreatment method should disrupt the complex matrix of the plant biomass, decrease the crystallinity of the cellulose, as well as limit the generation of inhibitory by-products and minimize hazardous wastes.

Ionic liquid (IL) is an organic salt with melting point below 100 °C. It is thermally stable, non-volatile, and can dissolve various polymeric compounds including cellulose and lignin under mild conditions [5, 6]. Many reports have shown that imidazolium IL, such as [Emim]Cl (1-ethyl-3-methylimidazolium chlorine), [Emim][OAc] (1-ethyl-3-methylimidazolium acetate), and [Bmim][OAc] (1-butyl-3-methylimidazolium acetate), is robust for lignocellulosic biomass pretreatment [7–9]. Compared with other conventional methods, IL pretreatment is promising due to its allowance to fractionate the biomass and obtain high purity cellulose, hemicellulose, and lignin fractions [10]. One of the challenges for large-scale application of IL pretreatment is the current high cost of IL [11]. The other challenge is that IL could affect the cell growth and product formation ability of industrial microorganisms [12–16]. For example, the presence of [Emim][OAc] (33.5–52.4 mM, depending on washing conditions) in corn stover hydrolysates showed strong inhibition of the cell growth and ethanol production by *Saccharomyces cerevisiae* [12]. In contrast, [Emim][OAc] (≤29.4 mM) and [Emim][DEP] (≤151.4 mM) enhanced ethanol production but also inhibited the growth of *S. cerevisiae* [15]. Recently, Dickinson et al. [16] found that [Emim]Cl exhibited its toxicity to *S. cerevisiae* by inducing abnormal mitochondrial morphology and hyperpolarized mitochondrial membrane potential; in addition, they speculated that hydrogen ion efflux might be coupled to influx of the toxic imidazolium cation. Huang et al. [17] studied the impact of [Emim][DEP] (1-ethyl-3-methylimidazolium diethyl phosphate), [Emim]Cl, and [Emim][OAc] on the cell growth and lipid accumulation of *Rhodosporidium toruloides*. They found that the inhibitory effect of [Emim][OAc] on lipid fermentation was due to the assimilation of acetate, which led to a rapid alkaline-pH shift of the medium. However, the mechanism with respect to the effect of imidazolium IL on cell growth and product formation of industrial microbes, especially for oleaginous microorganisms, remains largely unknown.

**Scheme 1:**
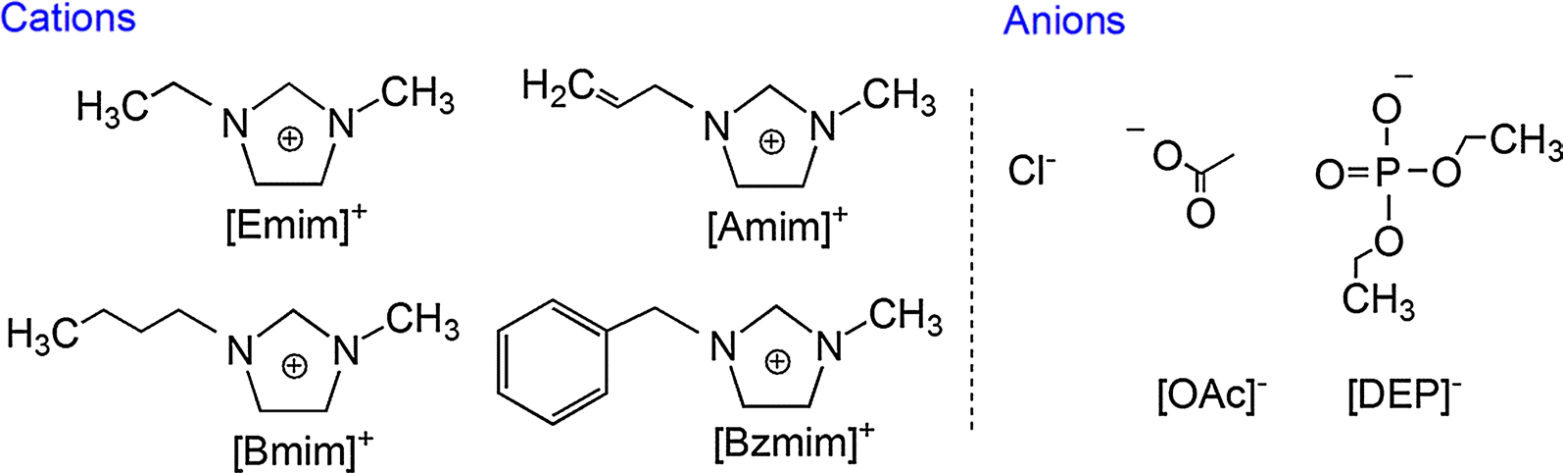
Structures of imidazolium ILs

*Geotrichum fermentans* CICC 1368, also known as *Trichosporon fermentans* CICC 1368, is an oleaginous yeast belonging to the family of Dipodascaceae that can accumulate lipids up to 65% of its dry weight on a variety of carbon sources [2, 18, 19]. More and more studies have shown that *G. fermentans* is an excellent lipid-producing yeast which can use various low-cost substrates, including waste molasses [2], rice straw hydrolysate [18], bagasse hydrolysate [20], and waste sweet potato vine hydrolysate [21] for lipid production with high efficiency. Besides, our previous studies found that the yeast exhibited a high tolerance to inhibitors and could even metabolize high concentrations of acetic acid [22–25]. In this study, the feasibility of *G. fermentans* using [Bmim][OAc]-pretreated rice straw hydrolysate as substrate for lipid production was explored for the first time. It is important to understand the effect of IL on the microbial lipid production to assess the possibility of using IL-pretreated lignocellulosic hydrolysates for biofuel production. Hence, seven imidazolium ILs (Scheme 1), including [Emim][DEP], [Emim]Cl, [Amim]Cl (1-allyl-3-methylimidazolium chlorine), [Bmim]Cl (1-butyl-3-methylimidazolium chlorine), [Bzmim]Cl (1-benzyl-3-methylimidazolium chlorine), [Emim][OAc], and [Bmim][OAc], which show robust pretreatment capacity for lignocellulosic biomass, were selected to study their impacts on the cell growth and lipid accumulation of *G. fermentans*. The influences of IL cation and anion were separately evaluated to make a clear correlation between the IL molecular structure and function. Sugar metabolism, the activities of key enzymes involved in the lipid synthesis, changes of cell membrane permeability, cell morphology, and cell surface topography of *G. fermentans* during lipid fermentation were systematically investigated to better understand the inhibitory mechanism of these imidazolium ILs. Our results will provide fundamental data needed if IL-based biomass pretreatment is to be incorporated into microbial oil production.

## Results and discussion

### Cell growth and lipid accumulation of *G. fermentans* on [Bmim][OAc]-pretreated rice straw hydrolysate

IL, such as [Bmim][OAc], can efficiently be used to pretreat lignocellulosic biomass at solid loadings above 30% (w/w) [8]. Here, the feasibility of [Bmim][OAc]-pretreated rice straw hydrolysate as a substrate for lipid production by *G. fermentans* was first evaluated.

The pretreatment conditions and enzymatic hydrolysis results are shown in Tables 1 and 2, respectively. Rice straw biomass yield and residual [Bmim][OAc] that remained in the enzymatic hydrolysates both decreased with the increase of washing steps. The decrease in plant biomass yield could be attributed to the loss of plant biomass and the removal of residual IL [12]. The highest hydrolysis yield of 66.1% was obtained in batch 2, corresponding to 28.1 g/L glucose and 9.2 g/L xylose. The residual [Bmim][OAc] that remained in the hydrolysate was 3.6% (wt/wt), which was close to the value (3.5%, wt/wt) reported by Li et al. [26]. A further increase in the washing step resulted in a slight drop in hydrolysis yield, which might be ascribed to the loss of plant biomass during the washing process.

**Table 1:** Pretreatment of rice straw with [Bmim][OAc]

Batch number	Weight % feedstock	T/^o^C	Precipitation solvent	Wash solvent	Biomass yield (wt%)	IL content (wt%)
1	33	135	1 × 10 g water	2 × 10 g water	90.1 ± 1.6	10.8 ± 0.3
2	33	135	1 × 10 g water	5 × 10 g water	82.7 ± 0.9	3.6 ± 0.3
3	33	135	1 × 10 g water	9 × 10 g water	80.6 ± 1.0	1.6 ± 0.1

**Table 2:** Material balance of biomass hydrolysis, sugar yields, and concentration of residual [Bmim]^+^ and acetate for regenerated rice straw

Batch number	Biomass loading (%)	Glucose (g/L)	Xylose (g/L)	Hydrolysis yield (%)	Acetate (g/L)	[Bmim]^+^ (g/L)
1	10	21.6 ± 0.4	7.3 ± 0.2	54.8 ± 1.1	5.1 ± 0.3	10.8 ± 0.7
2	10	28.1 ± 0.6	9.2 ± 0.3	66.1 ± 1.6	0.7 ± 0.1	3.6 ± 0.4
3	10	27.7 ± 0.9	9.4 ± 0.2	65.8 ± 1.7	0.2 ± 0.0	1.7 ± 0.0

The hydrolysate generated from batch 2 was selected as the carbon source for lipid fermentation by *G. fermentans*. Additive nutrients were added into the hydrolysate to form a hydrolysate-based medium (Table 3). To understand the impact of IL remaining in the hydrolysate on the cell growth and lipid accumulation of yeast, a simulated medium (synthetic medium) that had the same composition as that of the hydrolysate-based medium but without IL was used as the control (Table 3). Biomass and lipid content were measured after 3 days of fermentation, when the highest lipid yield was achieved in the simulated medium (see Additional file 1: Figure S1). *G. fermentans* could grow and accumulate lipid on hydrolysate-based medium (Table 3). However, the yeast biomass and lipid content obtained on the hydrolysate-based medium were much lower than those in the simulated medium (4.4 vs. 7.8 g/L and 37.8 vs. 52.6%, respectively). Ouellet et al. [12] studied the ethanol production by *S. cerevisiae* on the [Emim][OAc]-pretreated plant biomass hydrolysate and found that the residual [Emim][OAc] that remained in the hydrolysate was the primary source of inhibition of downstream microbial growth and ethanol formation. Therefore, the residual [Bmim][OAc] present in the rice straw hydrolysate might be responsible for the inhibitory effect on the lipid production by *G. fermentans*. Overall, the results demonstrated that *G. fermentans* can grow and accumulate lipid in the presence of IL.

**Table 3:** Lipid production by *G. fermentans* on hydrolysate-based medium and its simulated medium

Batch	Biomass (g/L)	Lipid content (%)	Lipid yield (g/L)	Lipid coefficient (%)	Relative fatty acid content (%)				
					C 16:0	C 18:0	C 18:1	C 18:2	Others
Simulated medium^a^	7.8 ± 0.6	52.6 ± 2.2	4.1 ± 0.5	17.7 ± 0.7	21.7 ± 0.2	11.3 ± 0.4	60.6 ± 0.5	5.3 ± 0.2	1.0 ± 0.1
Hydrolysate-based medium^b^	4.4 ± 0.1	37.8 ± 0.1	1.7 ± 0.0	7.5 ± 0.2	29.9 ± 0.3	9.1 ± 0.1	53.5 ± 1.0	6.7 ± 0.2	0.8 ± 0.0

^a^The simulated medium was used as the control and contained (g/L): glucose 28.14, xylose 9.21, peptone 0.714, yeast extract 0.255, MgSO_4_ 0.4, NaH_2_PO_4_ 2.0, MnSO_4_·7H_2_O 0.003, and CuSO_4_·5H_2_O 0.0001. The C/N molar ratio was 150

^b^The hydrolysate-based medium contained (g/L): rice straw hydrolysate, peptone 0.714, yeast extract 0.255, MgSO_4_ 0.4, NaH_2_PO_4_ 2.0, MnSO_4_·7H_2_O 0.003, and CuSO_4_·5H_2_O 0.0001. The C/N molar ratio was 150

### The effect of selected imidazolium IL on the cell growth and lipid accumulation of *G. fermentans*

**Fig. 1:**
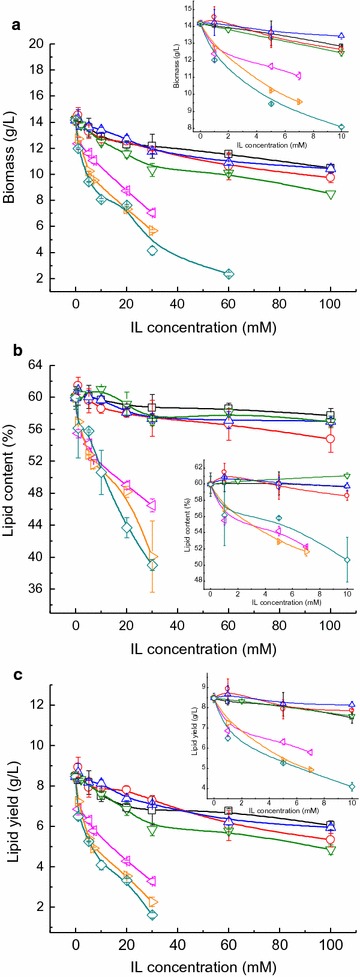
Effect of selected imidazolium IL on cell growth and lipid accumulation of *G. fermentans*. **a** Biomass, **b** lipid content, and **c** lipid yield. *Squares* [Emim][DEP], *circles* [Emim]Cl, *up triangles* [Amim]Cl, *down triangles* [Bmim]Cl, *left triangles* [Emim][OAc], *right triangles* [Bmim][OAc], *diamonds* [Bzmim]Cl. The fermentation medium contained (g/L): glucose 40, xylose 20, peptone 1.05, yeast extract 0.375, MgSO_4_ 0.4, NaH_2_PO_4_ 2.0, MnSO_4_·7H_2_O 0.003, CuSO_4_·5H_2_O 0.0001, and various concentrations of IL; the C/N molar ratio was 163. The medium without IL was used as the control

A series of imidazolium ILs with robust pretreatment capacity for lignocellulosic biomass, namely [Emim][DEP], [Emim]Cl, [Amim]Cl, [Bmim]Cl, [Bzmim]Cl, [Emim][OAc] and [Bmim][OAc], were added individually into the synthetic medium to systematically study their influence on the cell growth and lipid accumulation of *G. fermentans*. The inhibitory effects on the cell growth and lipid accumulation of *G. fermentans* were observed in all ILs tested as depicted in Fig. 1a and b. The biomass and lipid content decreased with an increase of IL concentration. Among the seven ILs tested, [Bzmim]Cl, [Emim][OAc], and [Bmim][OAc] showed the strongest inhibitory effects on the cell growth and lipid accumulation, and their inhibition potency showed the following order: [Bzmim]Cl > [Bmim][OAc] > [Emim][OAc]. For example, when 30 mM [Bzmim]Cl was supplemented, the biomass decreased from 14.2 to 4.2 g/L (corresponding to a 70.6% reduction) and the lipid content decreased from 60.0 to 39.0% (corresponding to a 34.9% reduction). Because of their high toxicity (no less than 50% inhibition of the lipid yield at 20 mM, Fig. 1c), it is necessary to either completely remove IL from hydrolysates or improve the resistance of microorganism strains to these ILs. The other four ILs showed mild inhibition of cell growth and lipid accumulation. Among the four ILs, [Emim][DEP] showed the weakest inhibition, which was in contrast to a previous study that showed that [Emim][DEP] exhibited greater suppression on lipid production by *R. toruloides* than [Emim]Cl [17], suggesting that toxicity of IL may be species dependent. Specifically, when exposed to 100 mM [Emim][DEP], the biomass, lipid content, and lipid yield of *G. fermentans* still remained 10.5 g/L, 57.7%, and 6.1 g/L, respectively, representing 74.0, 96.2 and 71.3%, respectively, of those of the control (fermentation in the absence of IL). The inhibition potency of the four ILs on lipid production showed the following order, [Bmim]Cl > [Emim]Cl ≈ [Amim]Cl > [Emim][DEP] (Fig. 1c). It is worth noting that the inhibition of the seven ILs on cell growth was stronger than on lipid accumulation. A similar phenomenon was also observed on studying the impact of organic acids present in lignocellulosic hydrolysates on lipid production by *G. fermentans* [22], demonstrating that the cell growth of the yeast was more susceptible to inhibitors than its lipid accumulation. Moreover, instead of inhibiting lipid production, some ILs even stimulated cell growth and/or lipid accumulation when supplemented at low concentrations. For example, [Emim]Cl and [Amim]Cl at a concentration of 1 mM could slightly stimulate cell growth and lipid accumulation, while [Emim][DEP] and [Bmim]Cl at low concentrations (≤5 mM for [Emim][DEP] and ≤10 mM for [Bmim]Cl) inhibited cell growth but enhanced lipid accumulation. Previous studies also demonstrated that IL at low concentrations could have a hormetic effect on microorganisms and human cell lines [27, 28]. However, the intrinsic mechanism of this hormetic effect still remains unknown.

Overall, the inhibition potency of the seven ILs on the lipid production showed the following order: [Bzmim]Cl > [Bmim][OAc] > [Emim][OAc] > [Bmim]Cl > [Emim]Cl ≈ [Amim]Cl > [Emim][DEP]. When the anion of IL was fixed (e.g., fixed to Cl^−^), it was found that the inhibition effects of different cations on cell growth and lipid accumulation were as follows: [Bzmim]^+^ > [Bmim]^+^ > [Emim]^+^ ≈ [Amim]^+^. When the cation of IL was fixed to [Emim]^+^, it was observed that the depression of different anions on lipid production showed the following order: [OAc]^−^ ≫ Cl^−^ > [DEP]^−^. Stolte et al. [29] also observed that the toxicity of imidazolium cation on environmental microorganisms increased with the increase of the length of its side chain. For [Bzmim]^+^, the strong inhibition of cell growth might be due to the toxicity of the benzyl group to microorganisms [30]. The phenomenon that [OAc]^−^ was more toxic than Cl^−^ was consistent with the results reported by other researchers [12, 17]. Moreover, Huang et al. found that [OAc]^−^ could be assimilated by *R. toruloides*, which led to an alkaline-pH shift of the medium [17]. We also measured the evolution of media pH and the concentration of [OAc]^−^ over time for cultivation in the presence of 30 mM [Emim][OAc] and [Bmim][OAc]. Similarly, the medium pH of the culture in the presence of [Emim][OAc] and [Bmim][OAc] was also increased compared with that of the culture in the absence of IL and the concentration of [OAc]^−^ decreased sharply after 1 day of cultivation, demonstrating that the anion of [Emim][OAc] and [Bmim][OAc] was also assimilated by *G. fermentans* (see Additional file 1: Figure S2). It was also reported that acetate could inhibit the phosphate uptake of yeast [31]. Thus, the high toxicity of [OAc]^−^ might be attributed to its capability to elevate medium pH after being assimilated and its inhibition of phosphate uptake by yeast.

In most cases, the addition of essential oils, plant extracts, organic acids, alcohol compounds, and IL could alter the fatty acid composition of microbial lipids [17, 32–34]. In this study, the effect of IL on the fatty acid composition of lipids produced by *G. fermentans* was also investigated. In all cases, oleic acid was the most abundant fatty acid, being 50–60% of the total fatty acids, followed by palmitic acid, stearic acid, and linoleic acid (see Additional file 1: Table S1).

[Emim][DEP], [Emim]Cl, [Amim]Cl, and [Bmim]Cl had no significant influence on the fatty acid composition of lipids over the range of concentrations tested (*p* > 0.05) except for [Emim][DEP] at 30 and 60 mM (significantly increased the relative content of linoleic acid, *p* < 0.05). On the other hand, [Bzmim]Cl, [Emim][OAc], and [Bmim][OAc] had little impact on the relative content of palmitic acid, but they decreased the relative content of stearic acid and oleic acid although this effect was significant only in the presence of 30 mM [Emim][OAc] (*p* < 0.05). In addition, except for [Bzmim]Cl at a concentration of 1 mM, the three ILs also increased the relative content of linoleic acid and this influence was significant in the presence of [Bzmim]Cl (≥10 mM, *p* < 0.05) and [Emim][OAc] (≥20 mM, *p* < 0.05). A similar influence on the relative content of oleic acid of lipids produced by *R. toruloides* was also observed by [Emim][OAc] and [Emim][DEP]; however, their impacts on the relative content of stearic acid and linoleic acid were different [17], demonstrating that the impact of IL on the fatty acid composition of lipid was species dependent.

### Effect of selected imidazolium IL on the sugar consumption by *G. fermentans*

The concentrations of glucose and xylose after 4 days of fermentation (when the lipid yield of the yeast in the absence of IL reached its maximum; data not shown) were examined to study the effect of selected IL on the sugar metabolism of *G. fermentans*.

**Fig. 2:**
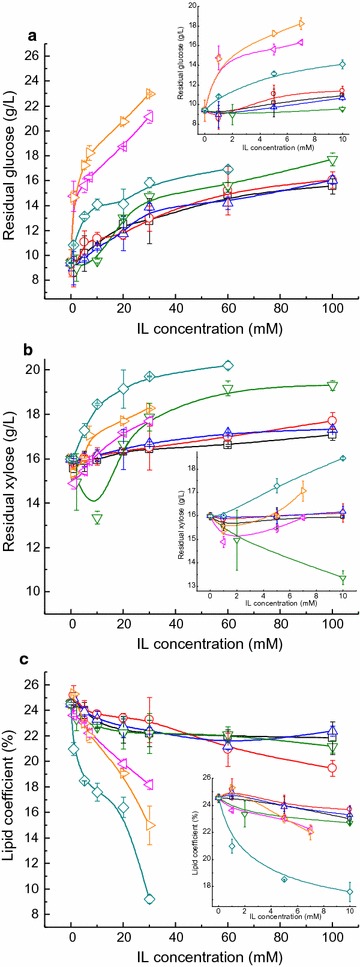
The effect of selected imidazolium IL on the sugar consumption and lipid coefficient of *G. fermentans*. **a** Glucose consumption, **b** xylose consumption, and **c** lipid coefficient. *Squares* [Emim][DEP], *circles* [Emim]Cl, *up triangles* [Amim]Cl, *down triangles* [Bmim]Cl, *left triangles* [Emim][OAc], *right triangles* [Bmim][OAc], *diamonds* [Bzmim]Cl. The fermentation medium contained (g/L): glucose 40, xylose 20, peptone 1.05, yeast extract 0.375, MgSO_4_ 0.4, NaH_2_PO_4_ 2.0, MnSO_4_·7H_2_O 0.003, CuSO_4_·5H_2_O 0.0001, and various concentration of IL; the C/N molar ratio was 163. The medium without IL was used as the control

Residual glucose and xylose in the absence of IL were 9.4 and 16.0 g/L, respectively (Fig. 2a). Interestingly, a small amount of IL, including [Emim][DEP], [Emim]Cl, [Amim]Cl, and [Bmim]Cl, especially [Bmim]Cl, could stimulate glucose and xylose consumption, which could well explain the promotive effect of these ILs on cell growth and/or lipid accumulation. Xylose utilization was also improved in the presence of 1 mM [Emim][OAc] and [Bmim][OAc] (Fig. 2b); however, no increase in the biomass or lipid content was observed, which might be due to the suppression of glucose consumption by these two ILs. At higher concentrations (≥10 mM), all the selected ILs inhibited glucose and xylose utilization, and the higher the concentration, the more pronounced was the inhibition, which was consistent with their influence on the cell growth and lipid accumulation of *G. fermentans*. The lipid coefficient in the presence of various ILs was also calculated (Fig. 2c). The lipid coefficient of *G. fermentans* in the presence of [Emim][OAc], [Bmim][OAc], and [Bzmim]Cl decreased sharply with the increase of IL concentration, whereas it dropped slightly when exposed to the other four ILs. Hence, the inhibition of sugar consumption by IL could partially explain its depression effect on the cell growth and lipid accumulation of *G. fermentans*.

### Effects of selected imidazolium IL on the key enzyme activities of *G. fermentans*

**Fig. 3:**
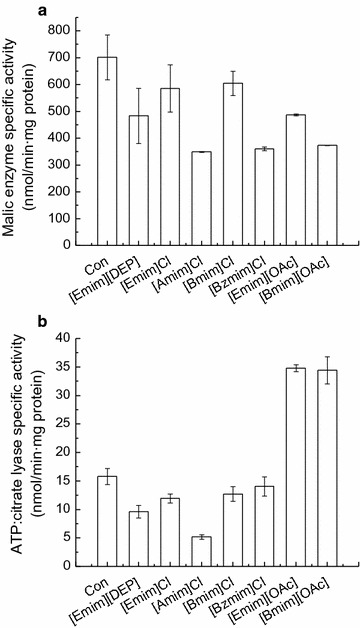
The effect of the selected imidazolium IL on (**a**) malic enzyme and (**b**) ATP-citrate lyase activities of *G. fermentans*. The fermentation medium contained (g/L): glucose 40, xylose 20, peptone 1.05, yeast extract 0.375, MgSO_4_ 0.4, NaH_2_PO_4_ 2.0, MnSO_4_·7H_2_O 0.003, CuSO_4_·5H_2_O 0.0001, and 30 mM IL. The C/N molar ratio was 163. The medium without IL was used as the control

A sufficient supply of NADPH (reducing force) is needed for the overproduction of lipid in microorganisms. It is proposed by Ratledge that malic enzyme (ME) is the major provider of NADPH for fatty acid biosynthesis [35]. Previous studies have demonstrated that the inhibitors present in dilute acid-hydrolyzed rice straw hydrolysate could inhibit the activity of ME in *G. fermentans* [22–24]. To obtain a deep insight into the impact of the selected imidazolium IL on the lipid production by *G. fermentans*, the activity of ME was detected after fermentation for 2 days, when the lipid accumulation rate reached the maximum in the medium without IL [22]. The results obtained above demonstrated that the concentration of residual [Bmim]^+^ in the rice straw hydrolysate of batch 2 was 25.6 mM (3.6 g/L); therefore, 30 mM was selected as the concentration of IL supplemented with the fermentation medium and this concentration was also used in subsequent studies. ME activity was inhibited by all ILs tested; it dropped from 701.4 nmol/(min mg protein) to 604.7–360.3 nmol/(min mg protein), which was consistent with the depression effect of these ILs on lipid accumulation (Fig. 3a). In addition to NADPH, acetyl-CoA as a precursor for fatty acid synthesis produced by ATP-citrate lyase (ACL) is another prerequisite for oleaginicity [36]. Similarly, the activity of ACL was also depressed by all ILs tested, except for [Emim][OAc] and [Bmim][OAc], which could partly explain the decreased lipid content of *G. fermentans* in the presence of these ILs (Fig. 3b). Interestingly, [Emim][OAc] and [Bmim][OAc] could promote the activity of ACL by 121 and 118%, respectively, compared with that of the control (34.8 and 34.4 vs. 15.8 nmol/min mg protein). It is likely that acetate could be assimilated and converted into acetyl-CoA, which improved the activity of ACL [37]. However, the lipid accumulation of *G. fermentans* exposed to [Emim][OAc] and [Bmim][OAc] was not enhanced, which might be due to the depressed ME activity. It was also reported that there was no correlation between the specific activity of ACL and the lipid content of a cell [36]. In general, the depression effect of imidazolium IL on key enzyme activities in fatty acid synthesis could partly explain its inhibition of lipid production by *G. fermentans*.

### Effect of selected imidazolium IL on the cell membrane integrity of *G. fermentans*

**Fig. 4:**
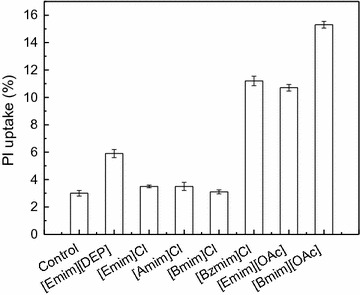
The effect of selected imidazolium IL on the cell membrane integrity of *G. fermentans*. The fermentation medium contained of (g/L): glucose 40, xylose 20, peptone 1.05, yeast extract 0.375, MgSO_4_ 0.4, NaH_2_PO_4_ 2.0, MnSO_4_·7H_2_O 0.003, CuSO_4_·5H_2_O 0.0001, and 30 mM IL. The C/N molar ratio was 163. The medium without IL was used as the control

A previous study demonstrated that 1-octyl-3-methylimidazolium chloride could increase the plasma membrane permeability of PC12 (rat pheochromocytoma) cells [38]. Cells in the absence or presence of selected IL were stained with PI and monitored by flow cytometry to investigate whether the selected imidazolium IL could influence the cell membrane integrity of *G. fermentans*. The PI uptake of *G. fermentans* after 1 day of fermentation is shown in Fig. 4. As depicted, [Amim]Cl, [Emim]Cl, and [Bmim]Cl had little impact on the cell membrane integrity, as the PI uptake of cells in the presence of these ILs was identical to that in the absence of IL (3%). Liu et al. observed that the effect of IL ([*C*_*n*_ mim]Cl, *n* ≥ 8) on the cellular membrane permeability of *Scenedesmus obliquus* increased with chain length, and IL with longer alkyl chain lengths tended to possess stronger lipophilicity, which increased the chance of contact with the lipid bilayer and hydrophobic proteins of the membrane [39]. However, no such relationship was found in our study. It is possible that the chain lengths of [Amim]Cl, [Emim]Cl, and [Bmim]Cl were too short to influence the cellular membrane permeability of *G. fermentans*. Although the inhibitory effect of [Emim][DEP] on lipid production by *G. fermentans* was weaker than the above three ILs, it caused stronger influence on cell membrane integrity. The PI uptake of *G. fermentans* in the presence of [Emim][OAc], [Bzmim]Cl, and [Bmim][OAc] was much higher than that of the control (fermentation in the absence of IL), which was consistent with their inhibitory effects on lipid production. As the fermentation continued, the influence of IL on the cell membrane integrity disappeared, as the PI uptake of the cells in the presence of IL after 2 and 3 days of cultivation was the same as that of the control (data not shown), indicating that *G. fermentans* adapted, to some extent, to IL. The above results demonstrated that the influence of IL on the cell growth and lipid accumulation of *G. fermentans* could be partly, but not completely, attributed to its influence on cell membrane integrity. In addition, comparing the impact of [Emim]Cl and [Bmim]Cl on cell membrane integrity with that of [Emim][DEP], [Emim][OAc], and [Bmim][OAc], it can be concluded that the anion of the selected IL plays a major role in determining its impact on the cell membrane integrity of *G. fermentans*. Moreover, the greater impact of [Bzmim]Cl on cell membrane integrity compared with that of [Bmim]Cl indicates that the functional group (benzyl group) on the cation can markedly influence the effect of IL on the cell membrane integrity.

### Effects of selected imidazolium IL on the cell morphology and cell surface topography of *G. fermentans*

**Fig. 5:**
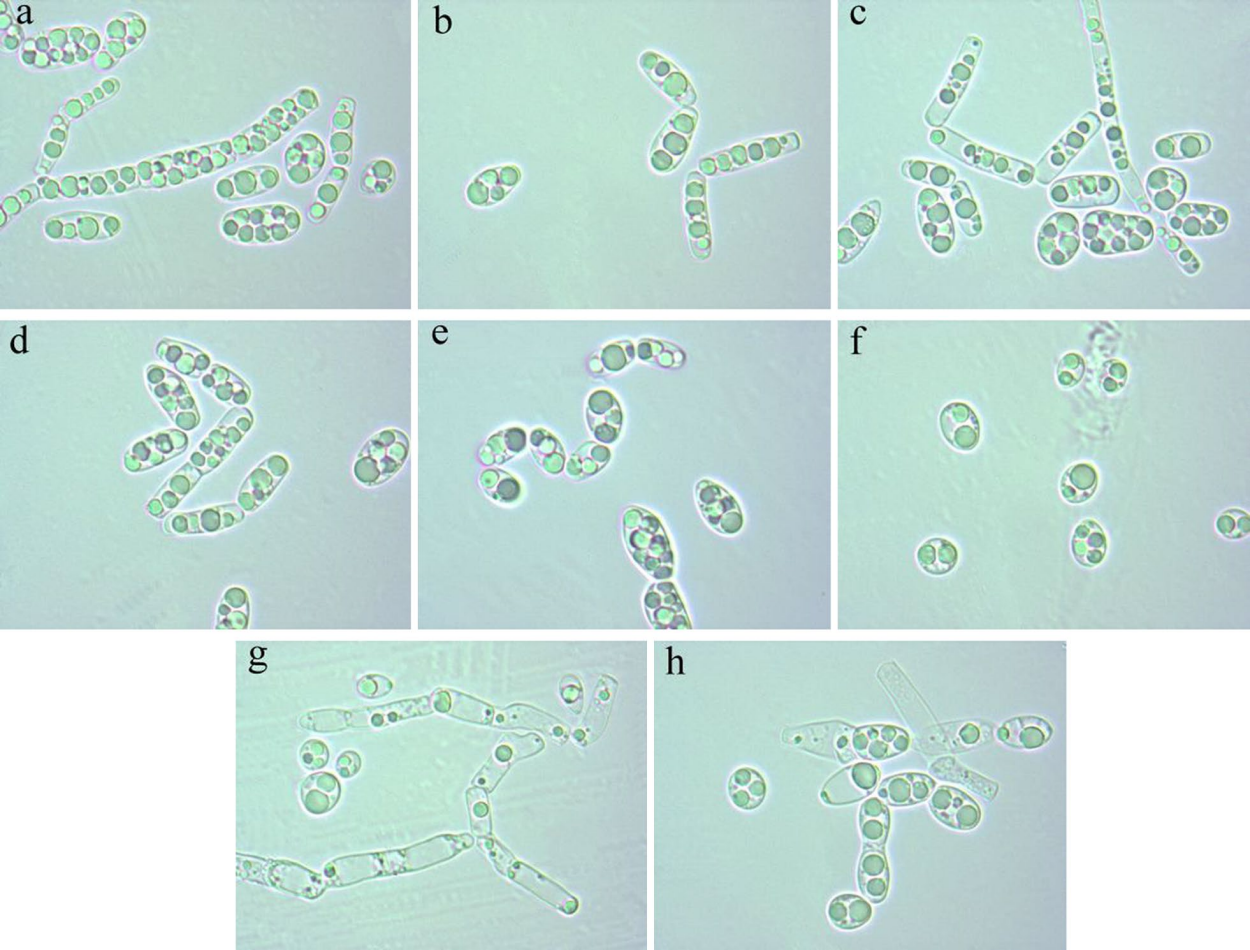
The cell morphology of *G. fermentans* after 4 days of fermentation in the presence of selected imidazolium IL. **a** Control, **b** [Emim][DEP], **c** [Emim]Cl, **d** [Amim]Cl, **e** [Bmim]Cl, **f** [Bzmim]Cl, **g** [Emim][OAc], and **h** [Bmim][OAc]. The fermentation medium contained (g/L): glucose 40, xylose 20, peptone 1.05, yeast extract 0.375, MgSO_4_ 0.4, NaH_2_PO_4_ 2.0, MnSO_4_·7H_2_O 0.003, CuSO_4_·5H_2_O 0.0001, and 30 mM IL. The C/N molar ratio was 163. The medium without IL was used as the control

The morphological changes of *G. fermentans* cultured in the media supplemented with 30 mM IL were also observed microscopically throughout the fermentation. Cells in the media with or without IL were mostly expanding hyphae after 1 day of fermentation (see Additional file 1: Figure S3). Then, they changed to ellipsoidal or oblong shape after 2–3 days of cultivation in the absence of IL or in the presence of [Emim][DEP], [Emim]Cl, [Amim]Cl, and [Emim][OAc]. However, in the presence of [Bmim]Cl, [Bzmim]Cl, and [Bmim][OAc], cells mostly changed to short ellipsoidal or subglobose shape. This phenomenon became obvious after 4 days of fermentation (Fig. 5). It seemed that [Bmim]Cl, [Bzmim]Cl, and [Bmim][OAc] could facilitate the formation of blastoconidia while suppressing the formation of arthroconidia of the yeast [40]. This was different from a previous study where there was no obvious relationship between the difference in cell morphology and the inhibition of alcohol compounds on the cell growth of *G. fermentans* [23].

**Fig. 6:**
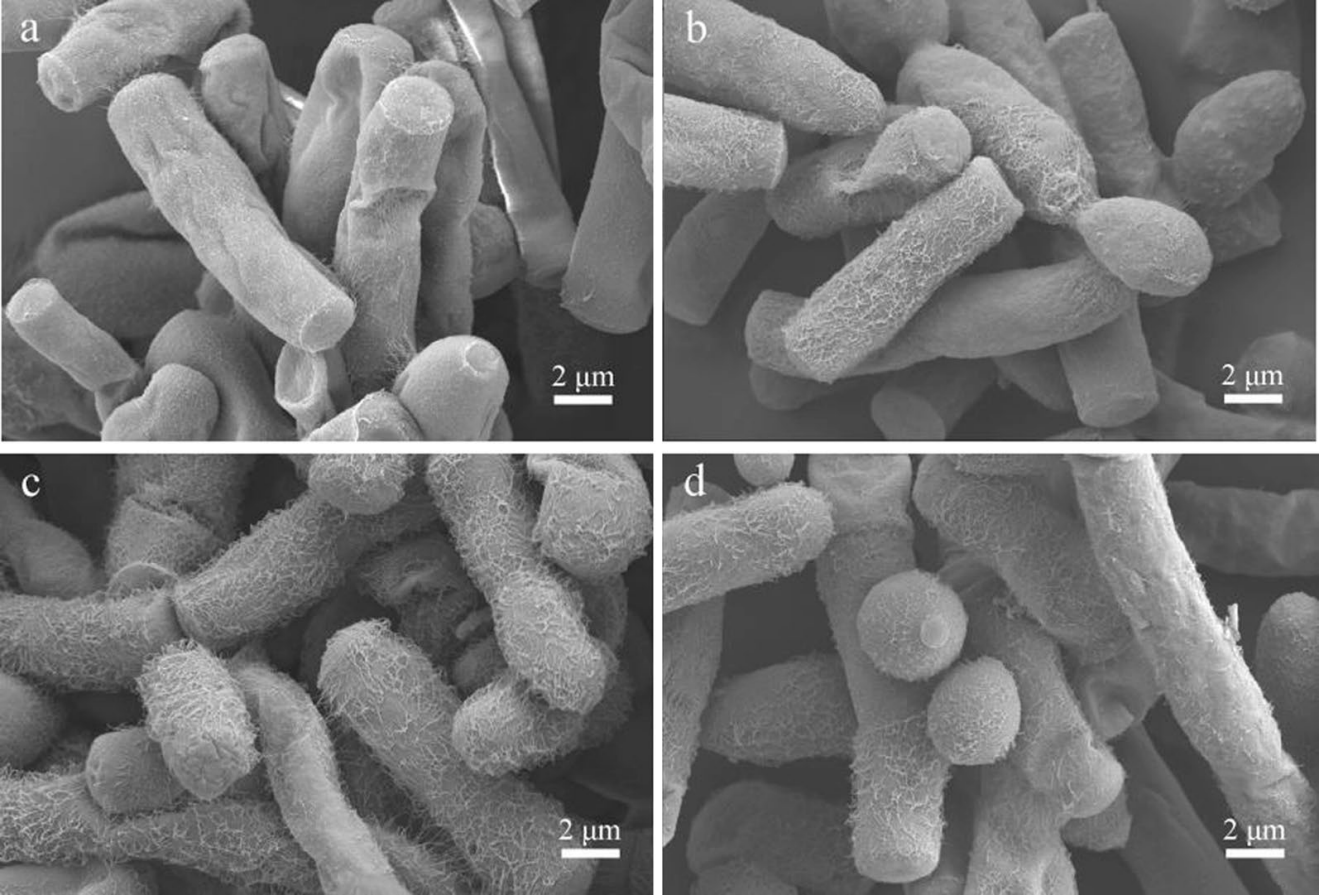
Effect of the selected imidazolium IL on the cell surface topography of *G. fermentans*. **a** Control, **b** [Bmim]Cl, **c** [Emim][OAc], and **d** [Bmim][OAc]. The fermentation medium contained (g/L): glucose 40, xylose 20, peptone 1.05, yeast extract 0.375, MgSO_4_ 0.4, NaH_2_PO_4_ 2.0, MnSO_4_·7H_2_O 0.003, CuSO_4_·5H_2_O 0.0001, and 30 mM IL. The C/N molar ratio was 163. The medium without IL was used as the control

The cell surface of a microorganism is in contact directly with the environment and may play an important role in adapting to the environment and protecting the cells. Cells incubated with IL that show superior lignocellulose pretreatment efficiency (i.e., [Bmim]Cl, [Emim][OAc], and [Bmim][OAc]) were visualized using SEM to further understand the impact of the selected imidazolium IL on the cell surface topography of *G. fermentans*. Samples were taken after 1 day of fermentation, since the cells seemed to be more susceptible at this time, as revealed by the results of our cell membrane integrity study. Cells in the absence of IL were mostly arthroconidia and the cell surface looked smooth (Fig. 6a). The surface topography of cells in the presence of [Bmim]Cl, [Emim][OAc], and [Bmim][OAc] are shown in Fig. 6b–d. Interestingly, the surface of the cells all seemed to be covered with fibrous structure. In addition, the fibrous structures on the surface of cells treated with [Emim][OAc] (Fig. 6c) and [Bmim][OAc] (Fig. 6d) were more abundant than those on the cells treated with [Bmim]Cl (Fig. 6b). The impact of [Emim][OAc] on the cell surface topography of *S. cerevisiae* was also studied by Mehmood et al., who found that after incubation with IL the cells exhibited wrinkled, softened, and holed shapes instead of forming fibrous structure on the cell surface [15]. It was also shown that *Phanerochaete chrysosporium,* when treated with IL, formed a biopolymer around the cells, which was composed of polysaccharides [41]. Hence, we speculate that the fibrous structure on the cell surface of *G. fermentans* exposed to IL is a biopolymer or a mucilaginous layer surrounding the cell wall for protecting the cells against IL [42]. However, the exact nature and function of the fibrous structure need to be further studied.

## Conclusions

The feasibility of using IL-pretreated rice straw hydrolysate for lipid production by *G. fermentans* was evaluated in this study. The results showed that *G. fermentans* could utilize [Bmim][OAc]-pretreated rice straw hydrolysate for cell growth and lipid accumulation; however, lipid production was suppressed in the presence of IL. A systematic study of seven imidazolium ILs, which were capable of efficient pretreatment of lignocellulosic biomass, revealed that [Emim][DEP], [Emim]Cl, [Amim]Cl, and [Bmim]Cl showed much less inhibition of the cell growth and lipid accumulation of *G. fermentans* than [Bzmim]Cl, [Emim][OAc], and [Bmim][OAc]. The IL cation showed a clear side chain effect, while the IL anion, [OAc]^−^ was more toxic than Cl^−^ and [DEP]^−^. Moreover, the seven ILs exerted their effects by inhibiting sugar metabolism and the activities of key enzymes involved in fatty acid synthesis. The relatively high toxicity of the three ILs ([Bzmim]Cl, [Emim][OAc], and [Bmim][OAc]) could be partly attributed to their influence on the cell membrane integrity of the yeast. SEM studies revealed that IL can induce fibrous structure on the cell surface, which might be an adaptive mechanism to IL. Our results will provide useful information in revealing the potential influence of imidazolium IL during its application in biorefinery processes. For IL with high toxicity, reducing its concentration in the subsequent hydrolysate or improving the tolerance of industrial microorganisms to IL is recommended.

## Methods

### Strain and chemicals

*Geotrichum fermentans* CICC 1368, also known as *T. fermentans* CICC 1368, was obtained from the China Center of Industrial Culture Collection and kept on Wort Agar (130 g/L malt extract, 15 g/L agar, and 0.1 g/L chloramphenicol) at 4 °C. 1-ethyl-3-methylimidazolium acetate ([Emim][OAc]) (>98.5%), 1-ethyl-3-methylimidazolium chlorine ([Emim]Cl) (>99%), 1-ethyl-3-methylimidazolium diethyl phosphate ([Emim][DEP]) (>99%), 1-allyl-3-methylimidazolium chlorine ([Amim]Cl) (>99%), 1-butyl-3-methylimidazolium acetate ([Bmim][OAc]) (>98.5%), 1-butyl-3-methylimidazolium chlorine ([Bmim]Cl) (>99%), and 1-benzyl-3-methylimidazolium chlorine ([Bzmim]Cl) (>99%) were purchased from Lanzhou Greenchem ILs, LIPC, CAS (Lanzhou, China) and used without any further purification. Cellulase cocktail (NS50013) and β-glucosidase (NS50010) were received courtesy of Novozymes (Denmark). Rice straw from Guangdong Province in Southern China was ground to less than 2 mm, vacuum dried at 60 °C for 24 h, and then stored in sealed containers at 4 °C before use. All other chemicals were reagent grade or better.

### Pretreatment of rice straw with [Bmim][OAc]

Pretreatment was performed according to the method of Wu et al. [8]. Rice straw biomass (1.0 g) was added to 2.0 g [Bmim][OAc] at room temperature in a 100 mL round bottom flask. After intensive mixing, the mixture was then placed in an oil bath and preheated to 135 ± 2 °C for 30 min and remained at the set point for 60 min. After heating, the heater was removed and 10 mL deionized water was added as an anti-solvent when the temperature of the mixture was cooled down to 50 °C. The precipitated rice straw biomass was filtered with 0.45 μm pore-size filter membrane under vacuum condition and washed with different amounts of water to investigate the impact of the washing process on the amount of residual IL in the subsequent hydrolysate (Table 1). After washing, the rice straw biomass was recovered by filtration and lyophilization (−70 °C and 0.25 mbar for 12 h; Christ ALPHA 2-4 LSC, German).

### Enzymatic hydrolysis

Enzymatic hydrolysis of [Bmim][OAc]-pretreated rice straw was carried out in 250 mL conical flasks with 30 mL of deionized water adjusted to pH 4.8 with 5 M H_2_SO_4_. The reactions used 10% total solid loading, and cellulase (NS50013) and β-glucosidase (NS50010) were added to a concentration of 90 and 9 mg protein/g glucan, respectively [12]. Hydrolysis was conducted at 50 °C with shaking at 200 rpm for 72 h. After hydrolysis, the hydrolysate was centrifuged at 12,000 rpm for 30 min, and the supernatant was stored at −20 °C for further study.

### Medium, precultivation, and cultivation

The precultivation medium (pH 6.0) contained (g/L) glucose 13.33, xylose 6.67, peptone 10, and yeast extract 10. The hydrolysate-based medium (pH 6.5) components contained rice straw hydrolysate, 0.714 g/L peptone, 0.255 g/L yeast extract, 0.4 g/L MgSO_4_, 2.0 g/L NaH_2_PO_4_, 0.003 g/L MnSO_4_·7H_2_O, and 0.0001 g/L CuSO_4_·5H_2_O. For comparison, the simulated medium containing the same components as those of hydrolysate-based medium except without IL was used as the control. The composition of the simulated medium was as follows (g/L): glucose 28.14, xylose 9.21, peptone 0.714, yeast extract 0.255, MgSO_4_ 0.4, NaH_2_PO_4_ 2.0, MnSO_4_·7H_2_O 0.003, CuSO_4_·5H_2_O 0.0001. To study the effects of various ILs on the lipid production by *G. fermentans*, cultivations were performed in a fermentation medium containing (g/L) glucose 40, xylose 20, peptone 1.05, yeast extract 0.375, MgSO_4_ 0.4, NaH_2_PO_4_ 2.0, MnSO_4_·7H_2_O 0.003, and CuSO_4_·5H_2_O 0.0001; IL at concentrations up to 60 or 100 mM was added into the fermentation medium before adjusting the medium pH to 6.5 with 5.0 M NaOH. Fermentation medium without any IL was used as the control. Media were sterilized at 115 °C for 20 min.

The preculture was performed in a 250 mL conical flask containing 50 mL of precultivation medium in a rotary shaker at 28 °C and 160 rpm for 24 h. For fermentation performed on hydrolysate-based medium and its control, 1.5 mL seed culture was inoculated into a 100 mL conical flask containing 28.5 mL hydrolysate-based medium or simulated medium. For cultivation performed on fermentation medium to examine the effect of IL, 2.5 mL seed culture was inoculated into a 250 mL conical flask containing 47.5 mL fermentation medium. All the fermentations were carried out in a rotary shaker at 25 °C and 160 rpm. For hydrolysate-based medium and its control, fermentation was performed for 3 days, while for fermentation medium it was performed for 4 days.

### Effects of selected IL on key enzymes activities and cell morphology of *G. fermentans*

Preparation of cell-free extracts and detection of malic enzyme activity were performed according to the method of Huang et al. [24] with a slight modification that sodium l-malate was replaced by l-malate. The activity of ATP-citrate lyase was assayed according to Huang et al. [43]. Protein concentrations were determined using the method of Bradford [44] with bovine serum albumin as a standard. The cell morphology of *G. fermentans* was observed under brightfield using an Axiostar Plus microscope (Zeiss, Germany) with an ocular lens at a 10× magnification and an oil lens at a 100× magnification.

### Flow cytometry

Propidium iodide (PI) binds to double-stranded nucleic acid and cannot cross an intact cytoplasmic membrane. Cells were collected by centrifugation (3000 rpm, 4 min), washed twice with phosphate-buffered saline (PBS) (0.1 M, pH 7.2), and then stained with PI (Key-GEN, China). Before analysis, cells were adjusted to approximately 10^6^ cells/mL and processed for sorting at a rate of approximately 500 events per second. In total, 10,000 events were detected in the reach run. Flow cytometry was carried out using a Cytomics™ FC500 (Beckman Coulter, UAS), and data were processed with Cytomics CXP software. PI was excited at 488 nm and measured at FL2 channel (575 nm).

### Scanning electron microscopy (SEM)

After 24 h of cultivation, cells were harvested by centrifugation at 3000 rpm for 4 min at 4 °C. Then, the cell pellets were washed twice with 0.1 M potassium buffer (pH 7.2) and pre-fixed in 2.5% glutaraldehyde for 12 h at 4 °C. Samples were subsequently washed three times with 0.1 M potassium buffer (pH 7.2), post-fixed in 1% osmium tetroxide for 30 min, then washed again with potassium buffer, and dehydrated in successive ethanol baths. After dehydration, the ethanol was replaced by isoamyl acetate and the cell samples were dried with CO_2_. Finally, samples were sputter coated with gold in an ion coater for 2 min, followed by microscopic examination (Merlin, Zeiss, Germany).

### Analytical methods

Yeast cell biomass was harvested by centrifugation, washed twice with distilled water, and dried at 105 °C for 24 h to get a constant weight. Extraction of lipid from dry cell biomass was performed according to the method of Bligh and Dyer [45] with some modifications. The biomass was soaked overnight in 4 M HCl and then heated in a boiling water bath for 10 min before being rapidly cooled at −20 °C. Lipid was extracted with a mixture of chloroform–methanol (2:1, v/v) for 1 h. After centrifugation, the chloroform phase was collected with a separatory funnel and removed by evaporation under vacuum at 55 °C and 100 rpm (NE-series rotary evaporator EYELA, Japan). Lipid content was defined as the ratio of lipid weight to biomass weight. Lipid yield was calculated as the amount of lipid extracted from the cells in per liter fermentation broth (g/L). Lipid coefficient was defined as grams of lipid produced per gram of sugar consumed and then multiplied by 100%.

The fatty acid profile of the lipid was determined as described by Morrison and Smith [46]. The fatty acid methyl esters produced by saponifying, followed by methylation of the lipid, were analyzed by gas chromatography (GC-2010, Shimadzu Corporation, Japan) with flame-ionization detector and a DB-Wax capillary column (30 m × 0.25 mm × 0.25 µm, Agilent Technologies Inc., USA). The column temperature was maintained at 180 °C for 2 min and then upgraded to 210 °C at a rate of 5 °C/min and kept for 11 min. Nitrogen was used as the carrier gas at 1.5 mL/min. The split ratio was 1:50 (v/v). The injector and the detector temperatures were set at 260 and 280 °C, respectively.

Glucose, xylose, and acetate were measured by HPLC as described by Huang et al. [18]. [Bmim]^+^ was analyzed by HPLC (Waters Crop., USA) using a UV detector at 210 nm (Waters 2489) and a Zorbax SB-C18 (Agilent Corp., USA), and the mixture of 10 mM NaCl water solution and acetonitrile (10/90, v/v) was used as the mobile phase at 1.0 mL/min. All reported data are the mean of duplicate experiments.

### Statistical analysis

All the experiments were performed at least in duplicate, and their average values with standard deviations were used for statistical analysis with SPSS 17.0 software for Windows (SPSS Statistics Inc., Chicago, IL, USA). One-way analysis of variance (ANOVA) and Tukey’s honestly significant differences (HSD) test were used to determine the significant differences of data at a 95% confidence interval.

## Additional file

**Additional file 1: Figure S1.** Cell growth and lipid accumulation of *G. fermentans* in simulated medium. The simulated medium contained (g/L): glucose 28.14, xylose 9.21, peptone 0.714, yeast extract 0.255, MgSO_4_ 0.4, NaH_2_PO_4_ 2.0, MnSO_4_·7H_2_O 0.003, CuSO_4_·5H_2_O 0.0001. The C/N molar ratio was 150. **Figure S2.** Variation of (a) pH and (b) [OAc]^-^ concentration during fermentation in the presence of IL. The fermentation medium contained (g/L): glucose 40, xylose 20, peptone 1.05, yeast extract 0.375, MgSO_4_ 0.4, NaH_2_PO_4_ 2.0, MnSO_4_·7H_2_O 0.003, CuSO_4_·5H_2_O 0.0001, and 30 mM IL. The C/N molar ratio was 163. The medium without IL was used as the control. **Table S1.** Effect of IL on fatty acid composition of lipid produced by *G. fermentans*. **Figure S3.** Cell morphology of *G. fermentans* in the presence of selected imidazolium IL. The fermentation medium contained (g/L): glucose 40, xylose 20, peptone 1.05, yeast extract 0.375, MgSO_4_ 0.4, NaH_2_PO_4_ 2.0, MnSO_4_·7H_2_O 0.003, CuSO_4_·5H_2_O 0.0001, and 30 mM IL. The C/N molar ratio was 163. The medium without IL was used as the control.

## Abbreviations

IL: ionic liquid
[Emim][DEP]: 1-ethyl-3-methylimidazolium diethyl phosphate
[Emim]Cl: 1-ethyl-3-methylimidazolium chlorine
[Amim]Cl: 1-allyl-3-methylimidazolium chlorine
[Bmim]Cl: 1-butyl-3-methylimidazolium chlorine
[Bzmim]Cl: 1-benzyl-3-methylimidazolium chlorine
[Emim][OAc]: 1-ethyl-3-methylimidazolium acetate
[Bmim][OAc]: 1-butyl-3-methylimidazolium acetate
ME: malic enzyme
ACL: ATP:citrate lyase
