# Supplementary material for: Mechanistic insights into the effect of imidazolium ionic liquid on lipid production by Geotrichum fermentans
Source: Biotechnol Biofuels. 2016 Dec 16;9:266. doi: 10.1186/s13068-016-0682-z (PMC5162095; doi:10.1186/s13068-016-0682-z)
Supplement: Supplementary file 1 — Additional file 1: Figure S1. Cell growth and lipid accumulation of G. fermentans in simulated medium. The simulated medium contained (g/L): glucose 28.14, xylose 9.21, peptone 0.714, yeast extract 0.255, MgSO4 0.4, NaH2PO4 2.0, MnSO4·7H2O 0.003, CuSO4·5H2O 0.0001. The C/N molar ratio was 150. Figure S2. Variation of (a) pH and (b) [OAc]- concentration during fermentation in the presence of IL. The fermentation medium contained (g/L): glucose 40, xylose 20, peptone 1.05, yeast extract 0.375, MgSO4 0.4, NaH2PO4 2.0, MnSO4·7H2O 0.003, CuSO4·5H2O 0.0001, and 30 mM IL. The C/N molar ratio was 163. The medium without IL was used as the control. Table S1. Effect of IL on fatty acid composition of lipid produced by G. fermentans. Figure S3. Cell morphology of G. fermentans in the presence of selected imidazolium IL. The fermentation medium contained (g/L): glucose 40, xylose 20, peptone 1.05, yeast extract 0.375, MgSO4 0.4, NaH2PO4 2.0, MnSO4·7H2O 0.003, CuSO4·5H2O 0.0001, and 30 mM IL. The C/N molar ratio was 163. The medium without IL was used as the control. [file 13068_2016_682_MOESM1_ESM.docx]

**Additional File 1**

**Mechanistic insights into the effect of imidazolium ionic liquid on the lipid production by *Geotrichum fermentans***

Li-ping Liu^2^, Min-hua Zong^1^, Robert J. Linhardt ^3^, Wen-yong Lou^1^, Ning Li^1^, Chao Huang^4^, Hong Wu^1,5,^*

^1^ School of Food Science and Engineering, South China University of Technology, Guangzhou 510640, China

^2^ School of Bioscience and Bioengineering, South China University of Technology, Guangzhou 510640, China

^3^ Department of Chemical and Biological Engineering, Rensselaer Polytechnic Institute, Troy, New York 12180, USA

^4^ Key Laboratory of Renewable Energy, Chinese Academy of Sciences, Guangzhou 510640, China.

^5^ Guangdong Province Key Laboratory for Green Processing of Natural Products and Product Safety, Guangzhou 510640, China

*Correspondence to Professor Hong Wu

Room 409, Building 13, South China University of Technology, 381 Wushan Rd., Tianhe District, Guangzhou 510640, China

E-mail address: bbhwu@scut.edu.cn

Tel: +86-20-22236669; Fax: +86-20-22236669

**Figure S1 Cell growth and lipid accumulation of *G. fermentans* in simulated medium.** The simulated medium contained (g/L): glucose 28.14, xylose 9.21, peptone 0.714, yeast extract 0.255, MgSO_4_ 0.4, NaH_2_PO_4_ 2.0, MnSO_4_·7H_2_O 0.003, CuSO_4_·5H_2_O 0.0001. The C/N molar ratio was 150.

**Figure S2 Variation of (a) pH and (b) [OAc]^-^ concentration during fermentation in the presence of IL.** The fermentation medium contained (g/L): glucose 40, xylose 20, peptone 1.05, yeast extract 0.375, MgSO_4_ 0.4, NaH_2_PO_4_ 2.0, MnSO_4_·7H_2_O 0.003, CuSO_4_·5H_2_O 0.0001, and 30 mM IL. The C/N molar ratio was 163. The medium without IL was used as the control.

**Table S1 Effect of IL on fatty acid composition of lipid produced by *G. fermentans*.**

| **Culture conditions** | **Relative fatty acid content (%)** | | | | |
| --- | --- | --- | --- | --- | --- |
|  | **C 16:0** | **C 18:0** | **C 18:1** | **C 18:2** | **Others** |
| Control |  |  |  |  |  |
| 0 mM | 21.7 ± 1.9 | 11.3 ± 2.0 | 60.6 ± 0.4 | 5.3 ± 0.1 | 1.0 ± 0.1 |
| [Emim][DEP] |  |  |  |  |  |
| 1 mM | 20.8 ± 1.3 | 11.4 ± 0.5 | 60.4 ± 0.7 | 5.7 ± 0.5 | 1.7 ± 0.1 |
| 5 mM | 21.2 ± 0.9 | 10.9 ± 0.2 | 59.9 ± 1.3 | 5.9 ± 0.3 | 2.1 ± 0.2 |
| 10 mM | 21.5 ± 1.1 | 11.6 ± 1.5 | 59.5 ± 2.1 | 6.0 ± 0.2 | 1.4 ± 0.0 |
| 20 mM | 20.7 ± 0.6 | 10.8 ± 1.2 | 61.5 ± 0.8 | 5.5 ± 0.6 | 1.6 ± 0.4 |
| 30 mM | 20.8 ± 1.6 | 9.9 ± 0.3 | 61.1 ± 1.5 | 7.0 ± 0.1 | 1.2 ± 0.2 |
| 60 mM | 20.7 ± 1.3 | 9.1 ± 0.1 | 60.0 ± 1.1 | 8.3 ± 0.4 | 2.1 ± 0.4 |
| 100 mM | 24.0 ± 1.0 | 8.9 ± 0.2 | 56.4 ± 1.8 | 7.0 ± 0.1 | 3.1 ± 0.6 |
| [Emim]Cl |  |  |  |  |  |
| 1 mM | 21.0 ± 0.5 | 11.8 ± 0.3 | 60.6 ± 2.1 | 5.3 ± 0.1 | 1.3 ± 0.2 |
| 5 mM | 19.5 ± 2.0 | 12.4 ± 0.6 | 61.5 ± 1.8 | 5.2 ± 0.4 | 1.4 ± 0.1 |
| 10 mM | 21.7 ± 0.9 | 11.7 ± 0.6 | 59.6 ± 0.5 | 5.5 ± 0.2 | 1.5 ± 0.0 |
| 20 mM | 21.1 ± 0.0 | 11.6 ± 0.1 | 60.5 ± 0.2 | 5.4 ± 0.0 | 1.4 ± 0.0 |
| 30 mM | 22.7 ± 1.1 | 10.6 ± 0.1 | 59.7 ± 1.1 | 5.5 ± 0.1 | 1.5 ± 0.0 |
| 60 mM | 22.6 ± 0.8 | 11.4 ± 0.5 | 58.9 ± 0.3 | 5.5 ± 0.0 | 1.4 ± 0.0 |
| 100 mM | 20.8 ± 0.1 | 11.4 ± 0.1 | 61.0 ± 0.4 | 5.5 ± 0.1 | 1.3 ± 0.2 |
| [Amim]Cl |  |  |  |  |  |
| 1 mM | 22.5 ± 0.1 | 11.1 ± 0.1 | 59.9 ± 0.0 | 5.6 ± 0.1 | 0.9 ± 0.0 |
| 5 mM | 22.9 ± 1.2 | 10.3 ± 0.2 | 60.0 ± 0.6 | 5.2 ± 0.3 | 1.5 ± 0.1 |
| 10 mM | 22.1 ± 0.3 | 11.8 ± 0.1 | 59.4 ± 0.5 | 5.3 ± 0.1 | 1.1 ± 0.2 |
| 20 mM | 22.8 ± 0.7 | 11.4 ± 0.4 | 59.4 ± 0.9 | 5.5 ± 0.1 | 0.9 ± 0.6 |
| 30 mM | 21.1 ± 0.3 | 11.6 ± 0.1 | 60.4 ± 0.0 | 5.4 ± 0.1 | 1.5 ± 0.0 |
| 60 mM | 22.6 ± 0.1 | 10.3 ± 0.9 | 60.6 ± 0.7 | 5.3 ± 0.2 | 1.2 ± 0.2 |
| 100 mM | 21.2 ± 0.2 | 10.7 ± 1.0 | 60.0 ± 1.3 | 5.4 ± 0.0 | 1.4 ± 0.6 |
| [Bmim]Cl |  |  |  |  |  |
| 2 mM | 22.4 ± 0.5 | 11.0 ± 0.1 | 59.4 ± 2.1 | 5.5 ± 0.3 | 1.6 ± 0.4 |
| 10 mM | 22.0 ± 0.2 | 11.8 ± 0.3 | 59.6 ± 0.9 | 5.2 ± 0.1 | 1.5 ± 0.1 |
| 20 mM | 22.8 ± 1.1 | 11.1 ± 0.0 | 59.9 ± 1.3 | 4.9 ± 0.4 | 1.4 ± 0.0 |
| 30 mM | 24.6 ± 0.2 | 8.3 ± 0.6 | 58.1 ± 0.3 | 7.3 ± 1.7 | 1.1 ± 0.2 |
| 60 mM | 24.7 ± 0.6 | 9.8 ± 0.7 | 58.9 ± 0.6 | 5.1 ± 1.1 | 1.5 ± 0.0 |
| [Bzmim]Cl |  |  |  |  |  |
| 1 mM | 21.4 ± 1.3 | 9.7 ± 0.8 | 59.4 ± 2.8 | 5.2 ± 0.2 | 4.3 ± 0.3 |
| 5 mM | 21.2 ± 0.6 | 9.0 ± 0.4 | 58.6 ± 2.2 | 7.3 ± 0.9 | 3.9 ± 1.1 |
| 10 mM | 22.5 ± 0.9 | 8.0 ± 0.1 | 56.0 ± 1.3 | 10.4 ± 0.4 | 3.1 ± 0.3 |
| 20 mM | 22.8 ± 0.8 | 7.0 ± 0.9 | 55.7 ± 2.0 | 11.2 ± 1.4 | 3.3 ± 0.5 |
| 30 mM | 23.1 ± 1.6 | 7.0 ± 0.3 | 55.0 ± 0.3 | 11.7 ± 0.7 | 2.2 ± 1.9 |
| [Emim][OAc] |  |  |  |  |  |
| 1 mM | 21.5 ± 0.6 | 10.0 ± 0.4 | 59.9 ± 0.0 | 6.8 ± 0.3 | 2.3 ± 1.1 |
| 5 mM | 21.4 ± 0.0 | 9.9 ± 0.2 | 58.9 ± 1.4 | 7.3 ± 0.3 | 1.1 ± 0.4 |
| 7 mM | 20.9 ± 0.7 | 9.9 ± 0.2 | 59.7 ± 0.6 | 7.7 ± 0.5 | 1.8 ± 0.6 |
| 20 mM | 22.7 ± 0.7 | 8.1 ± 0.3 | 55.5 ± 1.9 | 9.7 ± 0.7 | 3.9 ± 2.2 |
| 30 mM | 24.1 ± 0.7 | 7.7 ± 0.8 | 50.2 ± 1.6 | 12.3 ± 1.3 | 5.7 ± 0.3 |
| [Bmim][OAc] |  |  |  |  |  |
| 1 mM | 21.6 ± 0.7 | 8.8 ± 0.8 | 55.9 ± 4.6 | 10.2 ± 2.6 | 3.5 ± 2.0 |
| 5 mM | 22.3 ± 0.1 | 9.2 ± 0.1 | 58.2 ± 0.8 | 7.6 ± 0.1 | 2.7 ± 0.5 |
| 7 mM | 20.6 ± 1.2 | 8.7 ± 0.1 | 52.2 ± 2.0 | 9.7 ± 0.3 | 6.0 ± 0.2 |
| 20 mM | 25.1 ± 1.2 | 7.0 ± 0.5 | 51.0 ± 1.4 | 9.7 ± 1.2 | 4.1 ± 1.2 |
| 30 mM | 25.4 ± 1.2 | 6.7 ± 0.1 | 50.7 ± 2.0 | 11.8 ± 1.1 | 5.3 ± 2.2 |

The fermentation medium contained (g/L): glucose 40, xylose 20, peptone 1.05, yeast extract 0.375, MgSO_4_ 0.4, NaH_2_PO_4_ 2.0, MnSO_4_·7H_2_O 0.003, CuSO_4_·5H_2_O 0.0001, various concentration of IL, and the C/N molar ratio was 163. The medium without IL was used as the control.


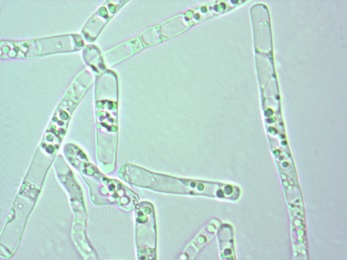

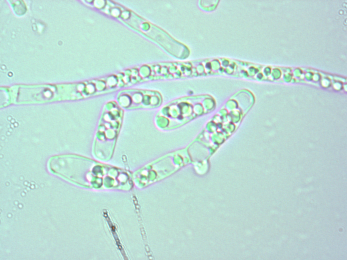

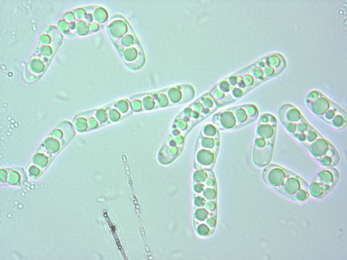


Control 1d Control 2d Control 3d


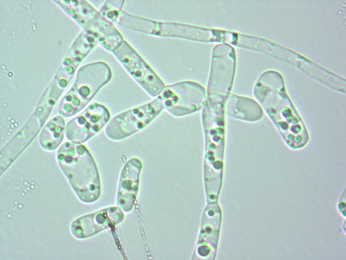

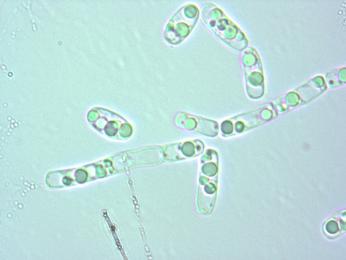

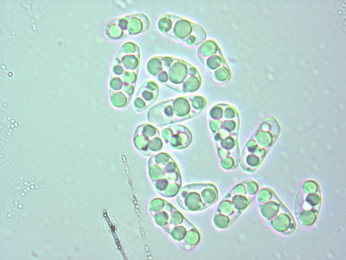


[Emim][DEP] 1d [Emim][DEP] 2d [Emim][DEP] 3d


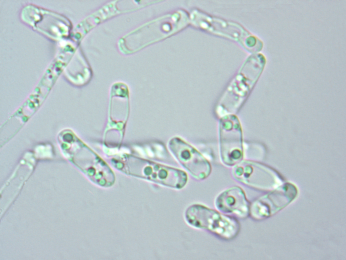

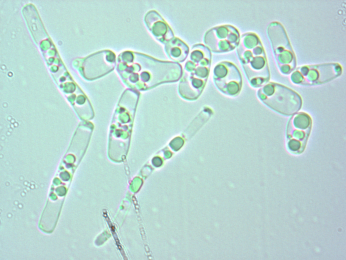

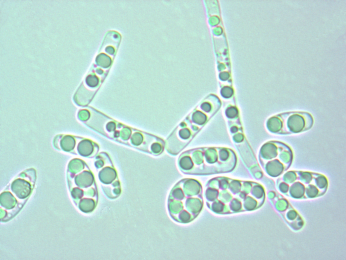


[Emim]Cl 1d [Emim]Cl 2d [Emim]Cl 3d


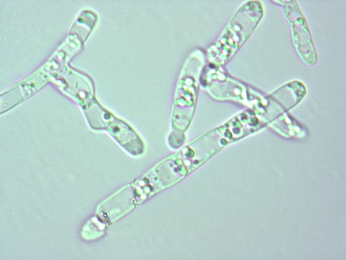

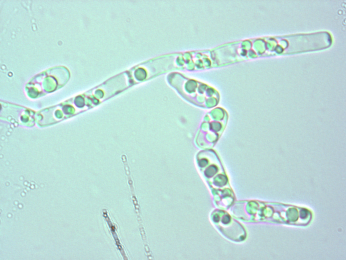

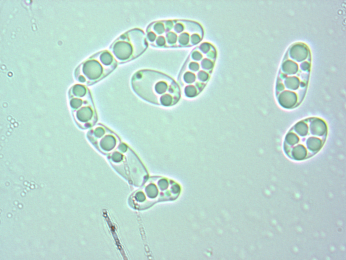


[Amim]Cl 1d [Amim]Cl 2d [Amim]Cl 3d


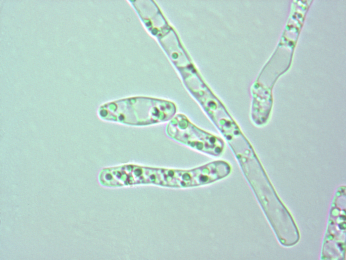

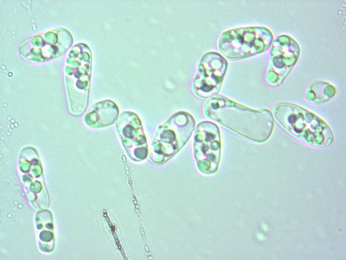

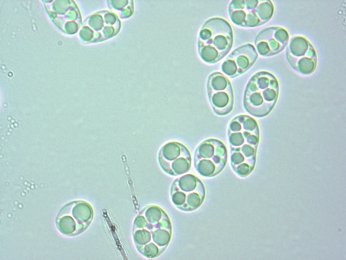


[Bmim]Cl 1d [Bmim]Cl 2d [Bmim]Cl 3d


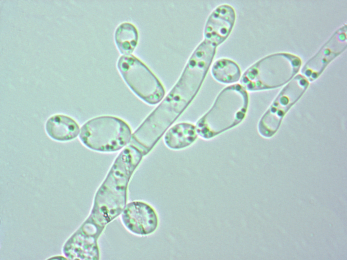

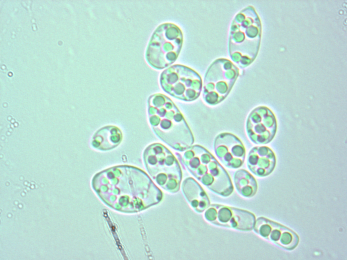

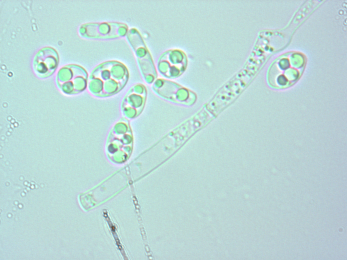


[Bzmim]Cl 1d [Bzmim]Cl 2d [Bzmim]Cl 3d


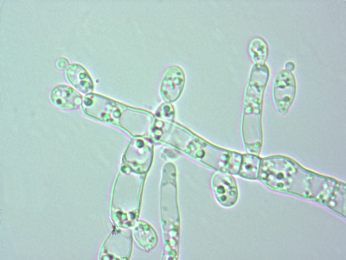

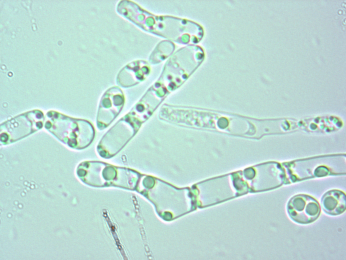

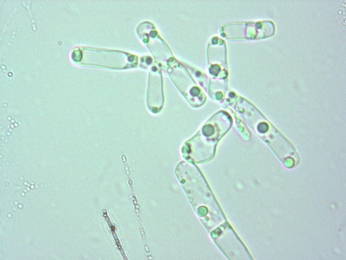


[Emim][OAc] 1d [Emim][OAc] 2d [Emim][OAc] 3d


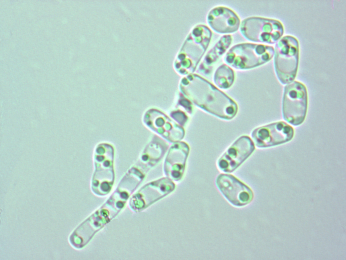

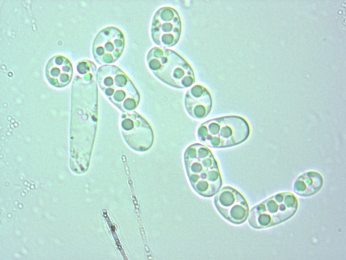

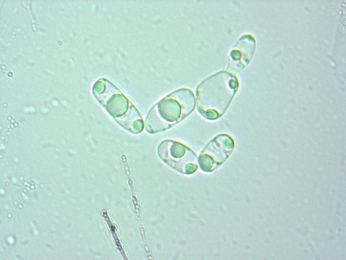


[Bmim][OAc] 1d [Bmim][OAc] 2d [Bmim][OAc] 3d

**Figure S3 Cell morphology of *G. fermentans* in the presence of selected imidazolium IL.** The fermentation medium contained (g/L): glucose 40, xylose 20, peptone 1.05, yeast extract 0.375, MgSO_4_ 0.4, NaH_2_PO_4_ 2.0, MnSO_4_·7H_2_O 0.003, CuSO_4_·5H_2_O 0.0001, and 30 mM IL. The C/N molar ratio was 163. The medium without IL was used as the control.
